# Testing Calibration of Cox Survival Models at Extremes of Event Risk

**DOI:** 10.3389/fgene.2018.00177

**Published:** 2018-05-22

**Authors:** David M. Soave, Lisa J. Strug

**Affiliations:** ^1^Program in Genetics and Genome Biology, Research Institute, The Hospital for Sick Children, Toronto, ON, Canada; ^2^Division of Biostatistics, Dalla Lana School of Public Health, University of Toronto, Toronto, ON, Canada; ^3^The Centre for Applied Genomics, The Hospital for Sick Children, Toronto, ON, Canada

**Keywords:** calibration tests, cox proportional hazards model, extreme risk, goodness-of-fit, prediction

## Abstract

Risk prediction models can translate genetic association findings for clinical decision-making. Most models are evaluated on their ability to discriminate, and the calibration of risk-prediction models is largely overlooked in applications. Models that demonstrate good discrimination in training datasets, if not properly calibrated to produce unbiased estimates of risk, can perform poorly in new patient populations. Poorly calibrated models arise due to missing covariates, such as genetic interactions that may be unknown or not measured. We demonstrate that models omitting interactions can lead to increased bias in predicted risk for patients at the tails of the risk distribution; i.e., those patients who are most likely to be affected by clinical decision making. We propose a new calibration test for Cox risk-prediction models that aggregates martingale residuals for subjects from extreme high and low risk groups with a test statistic maximum chosen by varying which risk groups are included in the extremes. To estimate the empirical significance of our test statistic, we simulate from a Gaussian distribution using the covariance matrix for the grouped sums of martingale residuals. Simulation shows the new test maintains control of type 1 error with improved power over a conventional goodness-of-fit test when risk prediction deviates at the tails of the risk distribution. We apply our method in the development of a prediction model for risk of cystic fibrosis-related diabetes. Our study highlights the importance of assessing calibration and discrimination in predictive modeling, and provides a complementary tool in the assessment of risk model calibration.

## 1. Introduction

Genome-wide association studies have been very successful in identifying genetic contributors to disease (Welter et al., 2014). Following discovery and validation, it is desirable to determine whether these genetic findings translate to biomarkers that can be clinically useful (e.g., for disease prognosis).

Two important measures of predictive performance of a risk-prediction model are discrimination and calibration (Harrell, 2001; Moons et al., 2012). Discrimination measures how well model-estimated risks translate to patient outcomes, where patients grouped according to higher predicted risk should demonstrate higher event rates than patients in lower risk groups. Calibration is a measure of how closely the estimated and observed absolute risks agree, where miscalibrated models lead to biased estimates of risk. Both measures are important for model validation in both training (internal) and external datasets, however, calibration is rarely reported in risk prediction studies (Collins et al., 2014). Even if a new prediction model discriminates well in a training dataset, if good calibration is not also achieved it can perform poorly in a new patient population. With the large cost and effort involved in obtaining external datasets for model validation, good model calibration should be demonstrated in the training set prior to collection of a second, independent sample.

The Cox proportional hazards (PH) model is a commonly used modeling technique for the analysis of time-to-event data. In the clinical setting, a Cox model can be used as a prediction tool to estimate an individual's relative (or absolute) risk of developing disease. Typically, a risk score is obtained as the linear predictor from the fitted Cox model. Patients can then be classified into risk groups to help inform clinical decisions. Methods to assess various aspects of the fit of a Cox model generally involve examination of plots of martingale residuals or their transforms (Schoenfeld, 1982; Barlow and Prentice, 1988; Therneau et al., 1990; Lin et al., 1993). Patterns in these plots can be challenging to identify in the presence of even moderate censoring, and thus, smoothers are typically applied as a visual aid. These smoothers can be useful in identifying trends, but give the impression of too little variation and therefore complementary formal testing is needed (Kalbfleisch and Prentice, 2002). Various calibration or goodness-of-fit (GOF) tests have been proposed in the Cox model setting, most of which can be characterized as variations of the Hosmer-Lemeshow GOF test for binary data (Hosmer et al., 1997). These methods assess the agreement between observed and expected risk across all risk levels, and therefore reflect a global assessment of lack of fit. Gronnesby and Borgan (1996) used counting process notation to derive a score (GB) test using the sums of martingale residuals across risk group deciles. The GB test is similar to the Hosmer-Lemeshow test since the martingale residuals correspond to the observed minus expected number of events for each subject. D'Agostino and Nam (2003) proposed a test comparing the average risk predictions with the observed Kaplan-Meier (K-M) failure probabilities across the deciles. This approach ignores censoring, however, leading to an incorrect variance estimate with increased instability for increased censoring (Crowson et al., 2016). Demler et al. (2015) proposed to use the robust Greenwood variance estimators of the K-M failure probabilities to improve performance of the testing procedure. While this approach maintains correct type 1 error control, it demonstrated comparable or lower power against the GB test under their simulation examples for model misspecification.

Clinical decisions about treatment and monitoring are most often made for patients at the extremes of the risk distribution (high or low). Therefore, accuracy of their predicted risks are a priority. While the available calibration tests for survival data have been shown to perform reasonably well as global tests, their power will be limited in detecting deviations in predicted risk at the extremes of the risk distribution where the proportion of subjects is small. Song et al. (2015) developed a method to test calibration of risk models at extremes of disease risk for binary outcomes. Their work was motivated by deviations between observed and expected risk near the tails of the risk distribution due to misspecification of either additive or multiplicative effects of the covariates on the risk. The Cox model also assumes that the effects of the covariates on the hazard rate (HR) are multiplicative, which may or may not be reasonable (Weinberg, 1986), and could result in bias in the expected hazard rate at the extremes of risk.

Genetic interaction (gene-gene and gene-environment) can contribute to complex traits. Many of these interactions remain unknown and are a challenge to model directly (Soave et al., 2015). Here we show that working models omitting relevant interactions are also likely to produce biased estimates of risk at the extreme tails of the population risk distribution.

Following the martingale theory used by Gronnesby and Borgan (1996), we propose a new calibration test for Cox models, that has improved power to detect biased risk estimates at the tails of the risk distribution. Our test aggregates martingale residuals for subjects from extreme high and low risk groups with a test statistic maximum chosen by varying which risk groups are included in the extremes. An estimate of the empirical significance of our test statistic is obtained by simulating from a Gaussian distribution using the covariance matrix for the grouped sums of martingale residuals. We describe and demonstrate how to implement our method using existing software. We conduct an extensive simulation study that shows the extreme risk (ER) test maintains good control of type 1 error and demonstrates improved power over the GB test when risk estimates are less accurate at the tails of the risk distribution. We consider scenarios where interaction effects are missing from the working model and the multiplicative risk assumption is violated. The ER test is complementary to existing global methods for examining risk model calibration.

## 2. Model and test procedures

For simplicity, we consider fixed time covariates, and right censoring of event times. For an independent sample of size *n*, let each individual *i* have a *p*x1 vector of fixed covariates, $z_{i}={\left(z_{i1},\ldots ,z_{ip}\right)}^{T}$. Let *T*_*i*_ and *C*_*i*_ be the event/failure time and censoring time, respectively for individual *i*, and only the earlier of the two times is observed. Following the counting process notation of Andersen et al. (1993), we observe *Y*_*i*_(*t*) and *N*_*i*_(*t*) at each time *t*, where *Y*_*i*_(*t*) = *I*(*T*_*i*_ ≥ *t, C*_*i*_ ≥ *t*) is the at risk indicator and *N*_*i*_(*t*) counts the number of observed events for individual *i* until time *t*. We assume that an event can occur only once for each individual. Thus, for each individual *i*, we observe a follow-up time *x*_*i*_ = *min*(*T*_*i*_, *C*_*i*_) and an indicator of whether an event occurred prior to censoring δ_*i*_ = *I*(*T*_*i*_ ≤ *C*_*i*_). Under the Cox PH model, the intensity process *h*_*i*_(*t*; ***z***_*i*_) for *N*_*i*_(*t*) can be written as

(1) $$\begin{array}{l} h_{i}\left(t;z_{i}\right)=h_{0}\left(t\right)exp\left(\beta^{T}z_{i}\right)Y_{i}\left(t\right), \end{array}$$

where *h*_0_(*t*) is the baseline hazard function, **β** is a *p*x1 vector of regression parameters and $\beta^{T}z_{i}$ is the risk score for individual *i*.

### 2.1. Gronnesby and borgan (GB) test

The GB test is based on martingale residuals, which are estimated for each individual at time *t* as

$$\begin{array}{l} {\hat{M}}_{i}\left(t\right)=N_{i}\left(t\right)-\int_{0}^{t}Y_{i}\left(u\right)exp\left({\hat{\beta }}^{T}z_{i}\right)d{\hat{\Lambda }}_{0}\left(u\right),i=1,\ldots ,n, \end{array}$$

where

$$\begin{array}{l} {\hat{\Lambda }}_{0}\left(u\right)=\int_{0}^{t}\frac{dN_{*}\left(u\right)}{\sum_{l=1}^{n}Y_{l}\left(u\right)exp\left({\hat{\beta }}^{T}z_{l}\right)} \end{array}$$

is the Breslow estimator (Breslow, 1972) of the baseline cumulative intensity process, and $N_{*}\left(u\right)=\overset{n}{\underset{i=1}{\sum }}N_{i}\left(t\right)$. We denote the estimated martingale at time *t* = ∞ as ${\hat{M}}_{i}\left(\infty \right)={\hat{M}}_{i}$.

For the GB test, the data are divided into *D* groups based on their estimated risk score, ${\hat{r}}_{i}={\hat{\beta }}^{T}z_{i}$, from the fitted Cox model of Equation (1). The martingale residuals are then summed within each group, $H_{J_{d}}=\underset{i}{\sum }K_{di}{\hat{M}}_{i}$, where $K_{di}=I\left({\hat{r}}_{i}\epsilon J_{d}\right)$ is an indicator for whether the risk score of the *i*th observation is in the risk score interval for the *d*th group, *J*_*d*_, *d* = 1, …, *D*. If the model fit is good [i.e., model (Equation 1) holds], *H*_*J*_*d*__ should be close to zero for each group, and $H={\left(H_{J_{1}},\ldots ,H_{J_{D-1}}\right)}^{T}$ converges to a mean zero multivariate Gaussian random vector (Gronnesby and Borgan, 1996). Therefore, the GB procedure uses the following test statistic:

$$\begin{array}{l} T=\left(H_{J_{1}},\ldots ,H_{J_{D-1}}\right){\hat{\Sigma }}^{-1}{\left(H_{J_{1}},\ldots ,H_{J_{D-1}}\right)}^{T}, \end{array}$$

where $\hat{\Sigma }$ is an estimate of the covariance matrix of ***H***. When model (Equation 1) holds, *T* is asymptotically distributed as $\chi_{D-1}^{2}$. Note that one of the group-wise martingale sums is omitted for model identifiability since ∑*H*_*J*_*d*__ = 0.

May and Hosmer (1998) showed that the GB test is algebraically equivalent to a score test of *D*−1 risk group indicator variables, ***K***_*i*_ = (*K*_1*i*_, …, *K*_(*D*−1)*i*_), in the Cox model (Equation 1). Thus, the GB test is equivalent to the following two-stage procedure:

Stage 1.1. Obtain the estimated risk score ${\hat{r}}_{i}$ for each subject from the Cox regression fit of model (Equation 1).
Stage 1.2. Divide the subjects into *D* groups based on the ordered risk score estimates, and specify group membership for each subject using the group indicator covariate vector, ***K***_*i*_.
Stage 2. Test for association with the indicator vector ***K***_*i*_ in the full model,
  (2) $$\begin{array}{l} h_{i}\left(t;z_{i},K_{i}\right)=h_{0}\left(t\right)exp\left(\beta^{T}z_{i}+\gamma^{T}K_{i}\right)Y_{i}\left(t\right). \end{array}$$

In this framework, the GB procedure is a score test of the null hypothesis that **γ** = **0**. The GB test now simplifies to fitting two standard Cox models with existing software.

### 2.2. Extreme risk (ER) test

The GB test is a global test of the model fit across the entire distribution of estimated risk scores. However, it may be desirable to focus additional attention at the extreme tails of the risk distribution where patients are more likely to be affected (positively or negatively) by clinical decisions. We propose a modification of the GB test to improve the power to detect model bias in risk prediction at the extremes of the risk distribution. This work is motivated by a recently proposed calibration test at extremes of disease risk for binary risk models (Song et al., 2015) and by recognition that risk-prediction models omitting relevant genetic interactions will increase bias in risk estimates at the extremes (section 3).

Again, suppose the data are divided evenly into *D* groups based on the estimated risk scores, as described above for the GB test. For a given pair of thresholds, *c* = (*c*_*l*_, *c*_*u*_), defining a set of “extreme” risk score groups, *R*_*c*_ = (*J*_1_, …, *J*_*c*_*l*__)∪(*J*_*c*_*u*__, …, *J*_*D*_), we propose the following test statistic

$$\begin{array}{l} T_{c}=\overset{D}{\underset{d=1}{\sum }}{\left(H_{J_{d}}\right)}^{2}I\left(J_{d}\epsilon R_{c}\right). \end{array}$$

This test statistic is observed to be the sum of the squared group martingales sums, over only those groups contained in the extreme risk set, *R*_*c*_. We do not incorporate the covariance matrix $\hat{\Sigma }$ in the definition of *T*_*c*_ but instead use it in a Monte Carlo simulation procedure as outlined below.

The motivation for the ER test arises from the departures detected at the tails of the risk distribution. However, specifying which groups should belong to the extreme risk set is arbitrary. The risk set should not be chosen by first looking at the data as this sort of adaptive procedure will lead to incorrect type 1 error control. Therefore, we propose taking our ER test statistic to be the maximum of a scaled version of *T*_*c*_, over all possible risk group sets (Song et al., 2015), $T^{max}=max_{c}\left({\tilde{T}}_{c}/n_{c}\right)$, where *n*_*c*_ is the number of groups included in *R*_*c*_ and ${\tilde{T}}_{c}$ is constructed using $\tilde{H}$, a scaled transformation of ***H*** such that each component has mean 0 and variance 1. In this way, *R*_*c*_ is chosen as a series of equally balanced groups beginning at both ends of the group list [i.e., *c* = (*c*_1_, *c*_*D*_), (*c*_2_, *c*_*D*−1_), (*c*_3_, *c*_*D*−2_), … etc.].

Under model (Equation 1), Gronnesby and Borgan (1996) derived explicit formulas for estimating the covariance matrix of $H,\hat{\Sigma }$. To achieve model identifiability, and estimate $\hat{\Sigma }$, the GB test arbitrarily omits the martingale sum for group *D*, *H*_*J*_*D*__, from ***H***. The ER test also requires estimation of $\hat{\Sigma }$, however, the focus is on detecting departures from the null hypothesis in the tails of the distribution. Thus we redefine ***H*** by omitting *H*_*J*_*D*/2__, when *D* is even, and *H*_*J*_(*D*+1)/2__, when *D* is odd, resulting in direct estimation of the covariance $\hat{\Sigma }$ for all groups except the median.

Next, ***H*** and $\hat{\Sigma }$ are scaled to be $\tilde{H}$ and $\tilde{\Sigma }$, such that $\tilde{\Sigma }$ is a correlation matrix. Unfortunately, the distribution of the test statistic *T*^*max*^ is intractable. However, with $\tilde{\Sigma }$ available, we can simulate realizations of $\tilde{H}$ (and correspondingly ${\tilde{T}}_{c}$ from each of the defined risk group sets, *R*_*c*_). Therefore, we propose the following steps to estimate the empirical *p*-value of the ER test, *P*(*T*^*max*^ ≥ *t*^*max*^), where *t*^*max*^ is the observed value of *T*^*max*^, using simulations as follows.

1. Generate a new realization of $\tilde{H}$ from a mean zero multivariate Gaussian distribution with covariance matrix $\tilde{\Sigma }$, and calculate a new value for the test statistic, $t_{s}^{max}$.
2. Repeat Step 1 *R* (*R*eplicate) times, to create a simulated “null" distribution for *T*^*max*^.
3. Estimate the *p*-value, *P*(*T*^*max*^ ≥ *t*^*max*^), empirically as the proportion of simulation replicates where the simulated $t_{s}^{max}$ is greater than the observed *t*^*max*^.

### 2.3. Implementing the ER test using existing software

Software packages generally estimate the regression coefficients, (**β**, **γ**), for a Cox model (Equation 2) by maximizing the log-partial likelihood

$$\begin{array}{l} \begin{array}{c} l\left(\beta ,\gamma \right)=\overset{n}{\underset{i=1}{\sum }}\delta_{i}[\beta^{T}z_{i}+\gamma^{T}K_{i}\\ -\;log\left(\overset{n}{\underset{l=1}{\sum }}exp\left(\beta^{T}z_{l}+\gamma^{T}K_{l}\right)Y_{l}\left(x_{i}\right)\right)]. \end{array} \end{array}$$

May and Hosmer (1998) showed that the partial likelihood score vector for **γ** under **γ** = **0** and $\beta ={\hat{\beta }}_{\gamma =0}$ corresponds to the vector of risk group sums of martingale residuals, ***H***. That is,

$$\begin{array}{l} \frac{\partial l\left(\beta ,\gamma \right)}{\partial \gamma }{|}_{\overset{\gamma =0}{\beta =\hat{\beta }}}=H. \end{array}$$

Therefore, we can extract an estimate of the covariance matrix of ***H*** from the observed information as

$$\begin{array}{l} \hat{\Sigma }=\left({\tilde{J}}_{\gamma \gamma }-{\tilde{J}}_{\gamma \beta }{\tilde{J}}_{\beta \beta }^{-1}{\tilde{J}}_{\beta \gamma }\right), \end{array}$$

where

$$\begin{array}{l} \tilde{J}=\begin{array}{c} (J_{\beta \beta }J_{\gamma \beta }\\ J_{\beta \gamma }J_{\gamma \gamma } \end{array}{|}_{\overset{\gamma =0}{\beta =\hat{\beta }}}\text{ and }J_{\beta \gamma }=\frac{\partial l\left(\beta ,\gamma \right)}{\partial \beta \partial \gamma^{T}}. \end{array}$$

Although not directly available as output from the coxph() function in the “survival" R software package (R Core Team, 2016), $\hat{\Sigma }$ can be obtained as follows (Web Appendix A in the Supplementary Materials provides example R code for this procedure).

1. Fit a Cox model corresponding to model (Equation 1) using coxph() to obtain estimates of the coefficients, $\hat{\beta }$.
2. Substitute these fixed estimates for **β** in a Cox model corresponding to model (Equation 2) while also specifying **γ** = 0; coefficients can be fixed in a coxph() fit by specifying “*iter.max=0”*.
3. Use the vcov() function to return the inverse of the observed information, $\tilde{I}$, and obtain $\hat{\Sigma }$ by taking the inverse of the submatrix with rows and columns corresponding to **γ**.

Obtaining $\hat{\Sigma }$ in this way allows one to avoid explicit specification of the formulas for $\hat{\Sigma }$ in Gronnesby and Borgan (1996). We can now simulate values of the grouped martingale sums, under the assumption of a correct model (Equation 1), and implement the ER test according to section 2.2.

### 2.4. Grouping strategy-choosing *D* and dealing with sparse vents within risk groups

Typically, a sample is stratified into 10 risk groups for the GB test. This convention is consistent with implementation of the Hosmer-Lemeshow test for binary data (Hosmer et al., 1997) and has been shown to yield good statistical properties for the GB test with samples sizes of 500 (May and Hosmer, 2004). We considered sample sizes of 5,000 and 1,500 in our simulation study, assuming that genetic markers individually contribute small effects to clinical outcomes. For implementation of both the GB and ER tests we use *D* = 11. The odd number of groups ensures that when the median group is omitted (as the reference group) from the simulation algorithm of the ER test, there is always a balanced number of upper and lower risk groups included in *T*_*c*_, for all values of the threshold, *c*.

A second convention for application of the Hosmer-Lemeshow test is the “no less than 5” events rule, directing that successive groups be collapsed based on a minimum of five expected events per group. Examination of this grouping convention for the GB test generally supports its application, although it may be conservative (May and Hosmer, 1998, 2004; Parzen and Lipsitz, 1999). For estimates of the expected number of events within the risk groups, we take the sum of the Cox-Snell residuals. The Cox-Snell residual corresponds to the second term in the martingale residual at *t* = ∞,

$$\begin{array}{l} \int_{0}^{\infty }Y_{i}\left(u\right)exp\left({\hat{\beta }}^{T}z_{i}\right)d{\hat{\Lambda }}_{0}\left(u\right),i=1,\ldots ,n. \end{array}$$

We compared our results with and without application of the ‘no less than 5’ events rule, and denote the corresponding GB and ER tests by GB_*adj*_ and ER_*adj*_.

## 3. Simulations

We conducted a simulation study to evaluate the performance of the ER test and compared it with the conventional GB test. To emulate calibration testing for risk-prediction models using genetic and environmental factors, we simulated 5 or 10 single nucleotide polymorphism (SNP) genotypes (*G* = 0, 1, or 2), one evnironmental exposure variable (*E* = 0, or 1), and event times (*t* in years) for each subject according to the Weibull hazard

(3) $$\begin{array}{l} h_{i}\left(t;G_{i}\right)=h_{0}\left(t\right)exp\left(g\left(\beta ,G_{i},E_{i}\right)\right), \end{array}$$

where $h_{0}\left(t\right)=\lambda \alpha t^{\alpha -1}$ is the baseline hazard, α is the shape parameter and λ is the scale parameter. The function *g*(·) specifies the model for the joint risk of the disease associated with the genotype-covariate vector ***G***_*i*_, exposure variable *E*_*i*_, and corresponding effect coefficients, **β**. We will use the notation *g*_0·_(·) and *g*_*A*·_(·) in (Equation 3) to specify various forms of the null (working) and alternative (true) hazard models, respectively. Each *G*_*ij*_ was simulated under Hardy-Weinberg equilibrium from a Binomial distribution to reflect the number of minor alleles (0,1,2) with minor allele frequency (MAF) 30% at each SNP *j* = 1, …, *p*, for each subject *i* = 1, …, *n*. All event times greater than 10 years were treated as censored at 10 years (administrative censoring). In addition, event times were uniformly censored prior to 10 years at a 0 or 50% censoring rate for different scenarios (lost to follow-up censoring). We considered sample sizes of *n* = 5,000 and 1,500. Type 1 error and power were assessed at the 0.05 significance level using 10,000 and 1,000 simulation replicates, respectively. To estimate the *p*-value for each ER test statistic, we used *R* = 1, 000 replicate simulations, which provided sufficient precision for the 0.05 significance threshold. Table 1 provides an outline of the simulation (and working) models used for power comparisons with the corresponding results figures. We chose to use small to moderate main effects for ***G*** that might be plausible for polygenic risk prediction models invovling complex traits. We also considered alternative effect sizes to those described in Table 1 and throughout the simulations, and obtained qualitatively similar comparisons between the ER and GB tests (results not shown).

**Table 1:** Outline of simulation study and results figures for power comparisons.

**Generating model**	**Working model**	**Parameter values**	**Results tables**	**Conclusions**
Missing Interactions (Categorical Covariates) - section 3.2				
	$g_{01}\left(\beta ,G_{i}\right)=\overset{p}{\underset{j=1}{\sum }}\beta_{G_{j}}G_{ij}$	*n* = 5,000, *p* = 10	Figure 2	ER showed increased power over GB for most models considered, however, GB showed a slight advantage for some of the larger interaction effect sizes.
		*n* = 1,500, *p* = 10	Web Figure 1 in the Supplementary Materials	
$g_{A1}\left(\beta ,G_{i},E_{i}\right)=\overset{p}{\underset{j=1}{\sum }}\beta_{G_{j}}G_{ij}$				
$+\beta_{E}E_{i}+\overset{p}{\underset{j=1}{\sum }}\beta_{G_{j}E}G_{ij}E_{i}$	$g_{02}\left(\beta ,G_{i},E_{i}\right)=\overset{p}{\underset{j=1}{\sum }}\beta_{G_{j}}G_{ij}+\beta_{E}E_{i}$	*n* = 5,000, *p* = 10	Figure 3	For data simulated under one interaction, ER was more powerful than GB. For data simulated under 10 interactions, the two tests performed comparably.
		*n* = 1,500, *p* = 10	Web Figure 2 in the Supplementary Materials	
Missing Interactions (Continuous Covariates) - section 3.2				
*g*_*A*3_(**β**, ***Z***_*i*_) = β_*Z*_1__*Z*_*i*1_+β_*Z*_2__*Z*_*i*2_	*g*_03_(**β**, ***Z***_*i*_) = β_*Z*_1__*Z*_*i*1_+β_*Z*_2__*Z*_*i*2_	*n* = 5,000, *p* = 10	Figure 4	ER demonstrated a noticeable power increase over GB.
+ β_*Z*_1_*Z*_2__*Z*_*i*1_*Z*_*i*2_		*n* = 1,500, *p* = 10	Not Shown	
Additive Effects - section 3.3				
$g_{A4}\left(\beta ,G_{i}\right)=log\left(1+\overset{p}{\underset{j=1}{\sum }}\beta_{G_{j}}G_{ij}\right)$	$g_{01}\left(\beta ,G_{i}\right)=\overset{p}{\underset{j=1}{\sum }}\beta_{G_{j}}G_{ij}$	*n* = 5,000, *p* = 5	Figure 5	ER was more powerful than the GB to detect departures from the multiplicative model.
		*n* = 5,000, *p* = 10	Web Figure 5 in the Supplementary Materials	

*Event times were simulated under the Weibull hazard model (Equation 3), $h_{i}\left(t;G_{i}\right)=\lambda \alpha t^{\alpha -\text{1}}exp\left(g\left(\beta ,G_{i}\right)\right)$ with a constant baseline hazard, α = 1, and λ chosen to provide a 20% event rate by time t = 10(years) in the absence of censoring. The function g(·) specifies the model for the joint risk of the disease associated with the genotype-covariate vector **G**_i_ and corresponding effect coefficients, **β**. Simulation results were grouped into figures by sample size (n) and number (p) of covariates (**G** or **Z**). See section 3 for complete simulation details*.

### 3.1. Type 1 error control of the ER test

To assess the type 1 error of our ER test, we simulated data from (Equation 3) under the null model (Equation 1) using $g_{01}\left(\beta ,G_{i}\right)=\overset{p}{\underset{j=1}{\sum }}\beta_{G_{j}}G_{ij}$, and then fit a corresponding Cox model of the same form. We used a fixed β_*G*_*j*__ across all SNPs of log(1.2) and log(1.15) for the models with *p* = 5 and 10 SNPs, respectively. We specified α at 1, 3 and 0.3, corresponding to a constant, increasing and decreasing baseline hazard, respectively. Under each scenario, λ was chosen such that the event rate prior to 10 years, in the absence of censoring, was 5%, 10% and 20%. For each simulation replicate we tested the Cox model for lack-of-fit using the ER and GB tests.

The proposed ER test maintained good control of type 1 error across all simulation scenarios for both the 5-SNP and 10-SNP models with constant, decreasing and increasing hazards (Table 2, Web Tables 1, 2 in the Supplementary Materials, respectively). Collapsing of groups based on the ‘less than 5’ expected events rule rarely occurred in the simulations with *n* = 5,000. For the simulations with *n* = 1,500, collapsing occurred more frequently, however, type 1 error results were similar for ER_*adj*_ and GB_*adj*_ (Web Table 2 in the Supplementary Materials) compared to the unadjusted ER and GB, respectively.

**Table 2:** Type-1 error of tests for constant baseline hazard.

**Event rate**	***p*** = **5**				***p*** = **10**			
	**0% censoring**		**50% censoring**		**0% censoring**		**50% censoring**	
	**GB**	**ER**	**GB**	**ER**	**GB**	**ER**	**GB**	**ER**
*n* = 5,000								
0.05	0.049	0.048	0.050	0.049	0.053	0.056	0.054	0.046
0.1	0.050	0.052	0.053	0.050	0.054	0.052	0.052	0.054
0.2	0.052	0.049	0.052	0.051	0.048	0.049	0.052	0.05
*n* = 1,500								
0.05	0.050	0.046	0.054	0.052	0.049	0.046	0.053	0.052
0.1	0.051	0.052	0.048	0.045	0.053	0.049	0.051	0.050
0.2	0.051	0.050	0.052	0.052	0.055	0.052	0.057	0.054

*Event times simulated under the Weibull hazard model (Equation 3) with g_01_ for a constant baseline hazard, α = 1, with p = 5 or 10 covariates (genotypes). Empirical size is presented for the Gronnesby and Borgen test (GB) and the proposed Extreme Risk test (ER). The empirical type 1 error was estimated from 10,000 simulated replicates at the nominal 5% level*.

Traditional application of the GB test compares the test statistic (*T* in section 2.1) to the $\chi_{D-1}^{2}$ distribution to obtain a *p*-value. Simulation based *p*-value estimates for GB similarly to the ER test method are also possible. We compared the type 1 error and power between the asymptotic and simulation based methods for GB and found the results to be very similar. Thus, we report only the results of GB based on the asymptotic *p*-value estimates.

### 3.2. Power of the ER test under misspecified models-missing interactions

To examine the power of the ER test to detect model misspecification due to missing interactions, we simulated data from (Equation 3) with the addition of an exposure, *E*, that interacted with one or more of the covariates, $g_{A1}\left(\beta ,G_{i},E_{i}\right)=\overset{p}{\underset{j=1}{\sum }}\beta_{G_{j}}G_{ij}+\beta_{E}E_{i}+\overset{p}{\underset{j=1}{\sum }}\beta_{G_{j}E}G_{ij}E_{i}$. We then fit a Cox model corresponding to (Equation 3) that included the main effect of *E* but omitted the interactions, $g_{02}\left(\beta ,G_{i},E_{i}\right)=\overset{p}{\underset{j=1}{\sum }}\beta_{G_{j}}G_{ij}+\beta_{E}E_{i}$, as well as one that completely ignored the presence of the exposure, *g*_01_(**β**, ***G***_*i*_). This may be a common occurrence as interacting exposures are often unknown to researchers. Each *E*_*i*_ was a binary (0,1) variable simulated from a Bernoulli distribution with frequency 0.3; smaller frequencies were also evaluated (0.2 and 0.1) but led to similar conclusions (results not shown). We used a fixed β_*G*_*j*__ of log(1.15) across all SNPs for the model with *p* = 10 SNPs. The main effect of the exposure, β_*E*_, was also fixed at the same value as the β_*G*_*j*__. We specified α at 1, corresponding to a constant baseline hazard. Under each scenario, the event rate prior to 10 years was 20% in the absence of censoring. To examine the empirical power, we varied a single exposure effect common to all interactions in the model, β_*G*_*j*_*E*_, across all scenarios.

**Figure 1:**
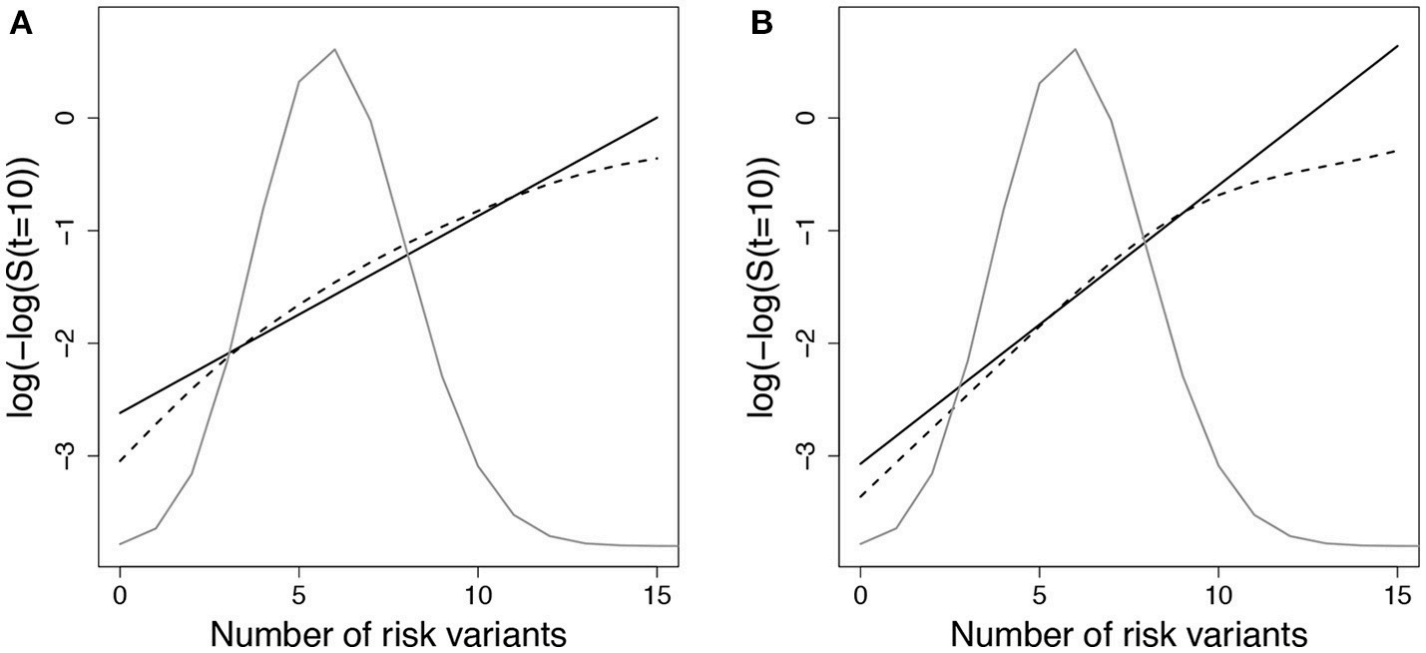
Log-log survival probability [*t* = 10 years] versus cumulative number of risk variants under missing interaction models. Time to event data were simulated under model (Equation 3) using *g*_*A*1_ with 10 SNPs (MAF=0.3 for each) and **(A)** one interaction with *E* ($g_{A1}\left(\beta ,G_{i},E_{i}\right)=\overset{10}{\underset{j=1}{\sum }}1.15G_{ij}+1.15E_{i}+10G_{i1}E_{i}$) or **(B)** ten interactions with *E* ($g_{A1}\left(\beta ,G_{i},E_{i}\right)=\overset{10}{\underset{j=1}{\sum }}1.15G_{ij}+1.15E_{i}+\overset{10}{\underset{j=1}{\sum }}1.35G_{ij}E_{i}$). Cox models were fit using the null model $g_{02}\left(\beta ,G_{i},E_{i}\right)=\overset{10}{\underset{j=1}{\sum }}\beta_{G_{j}}G_{ij}+\beta_{E}E_{i}$ to estimate survival probabilities at time *t*=10 years ($\hat{S_{i}}\left(t\right)=\hat{S_{0}}{\left(t\right)}^{exp\left(\overset{10}{\underset{j=1}{\sum }}{\hat{\beta }}_{G_{j}}G_{ij}+{\hat{\beta }}_{E}E_{i}\right)}$), which were then averaged by risk group (defined by cumulative number of risk variants) (solid line - predicted survival). Observed survival probabilities (dashed line - observed survival) were obtained from a Cox model comparing patients in each risk group with the reference group. The reference group was the subset of individuals with 6 risk alleles; the mean number of risk alleles from the 10-SNP model. The distribution of subjects per number of risk alleles is shown as a density curve. Survival probabilities estimated for other time points (*t* < 10 years) yielded similar results.

We expected that data simulated under *g*_*A*1_ and fit using a Cox PH model with *g*_01_ or *g*_02_ would show increased deviation in observed vs. expected risk at the extremes of the subject risk distribution. Figure 1 demonstrates the deviation between observed and predicted survival probabilities across the population risk distribution for the missing interaction scenario.

**Figure 2:**
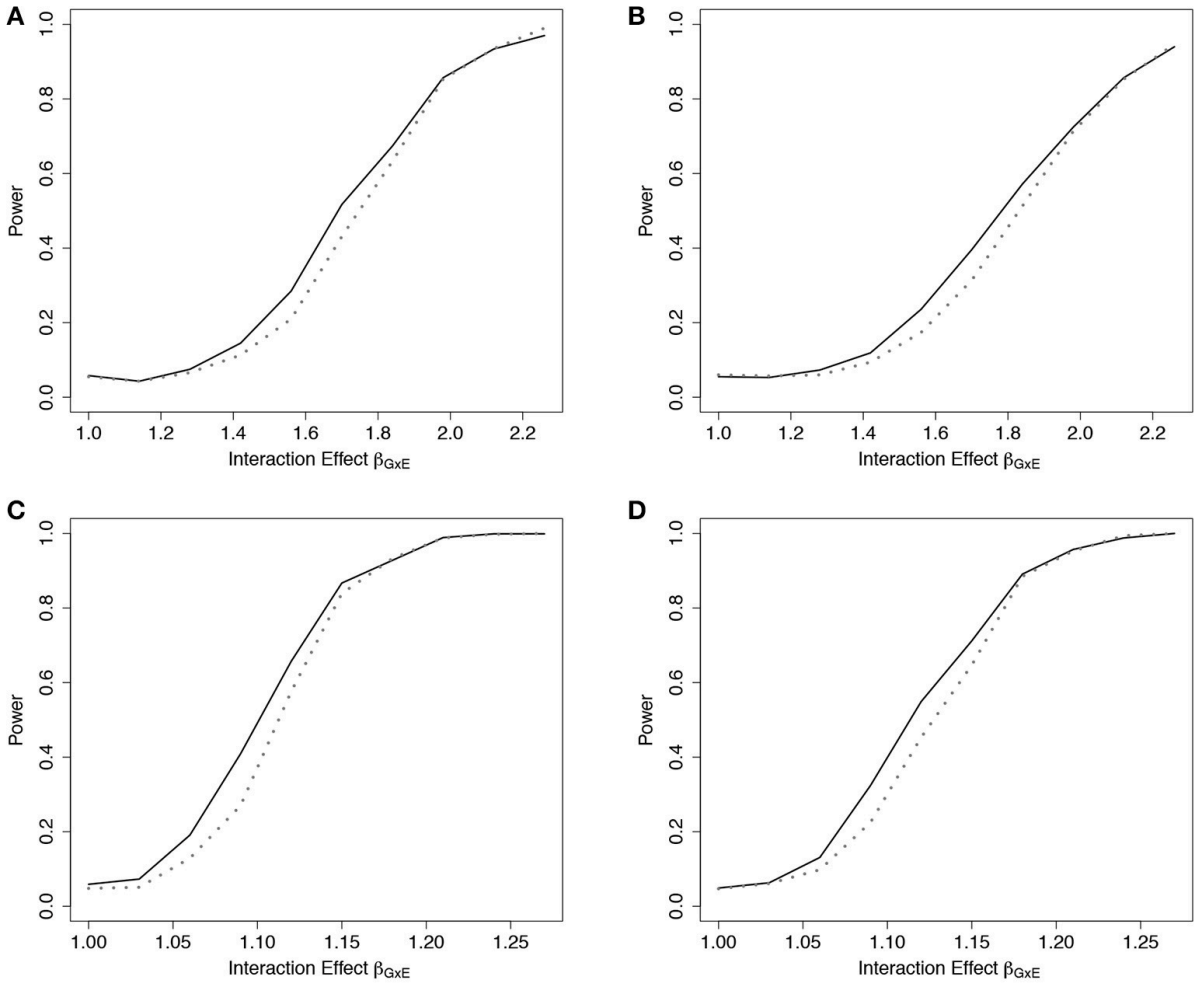
Power of tests under missing interaction models *g*_02_. Power of the ER (solid line) and GB (dotted line) tests when ***G***x*E* interaction terms are missing from the fitted Cox model (3), but exposure *E* is included, using *g*_02_. *n* = 5000 for the 10-SNP model with one interaction **(A**,**B)** or ten interactions **(C**,**D)** in the underlying true model *g*_*A*1_, and either 0% **(A**,**C)** or 50% **(B**,**D)** lost to follow-up censoring.

Empirical power results from the fitted models that included the main effect of *E* but omitted the interaction effect(s), *g*_02_, are presented in Figure 2 (*n* = 5,000) and Web Figure 1 in the Supplementary Materials (*n* = 1, 500) for the data simulated with 1 and 10 interactions between ***G*** and *E*. For both scenarios, the power of the ER and GB tests increased as the size of the omitted interactions increased. The ER test appeared to show an increase in power over GB for both the single and multiple (10) SNP-interaction models with 0% or 50% lost to follow up in the samples, for much of the range of the interaction effect, β_*G*_*j*_*E*_. The GB test showed a slight advantage for some of the larger interaction effect sizes considered.

**Figure 3:**
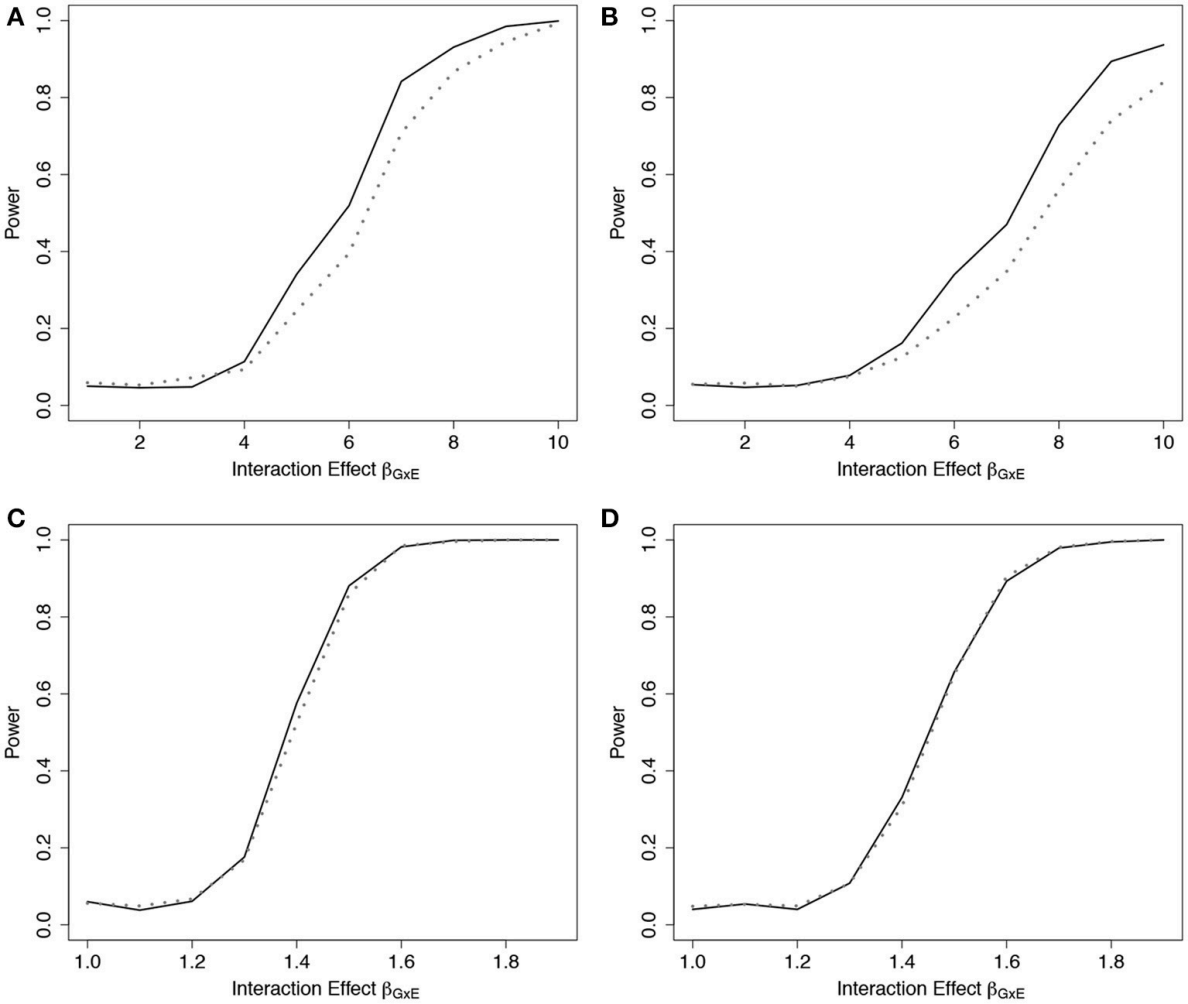
Power of tests under missing interaction models *g*_01_. Power of the ER (solid line) and GB (dotted line) tests when main effect of *E* and ***G***x*E* interaction terms are missing from the fitted Cox model (3), using *g*_01_. *n* = 5,000 for the 10-SNP model with one interaction **(A**,**B)** or ten interactions **(C**,**D)** in the underlying true model *g*_*A*1_, and either 0% **(A**,**C)** or 50% **(B**,**D)** lost to follow-up censoring.

Results from the fitted models that completely omitted *E* (both main effect and interactions), *g*_01_, are presented in Figure 3 (*n* = 5, 000) and Web Figure 2 in the Supplementary Materials (*n* = 1,500). For data simulated from models including one interaction between ***G*** and *E*, ER was more powerful than GB. For the data simulated under 10 interactions, the performance of the two tests was comparable. When one large interaction effect exists (say for *G*_1_·*E*) but is omitted in the fitted model along with the main effect *E*, the estimated effect on risk due to *G*_1_ = 1 will be larger (much larger in the case of *G*_1_ = 2) than the estimated effect of the other SNPÕs. Depending on the minor allele frequency of *G*_1_, individuals with either *G*_1_ = 0 or *G*_1_ = 2 will be over represented in either the lowest- or highest-risk groups, respectively, and contribute less stable martingale residuals to their risk group (larger in absolute value). On the other hand, when smaller interaction effects exist for each SNP, but are omitted, the bias that is introduced is likely to be spread across more risk groups, reducing the improvement observed for the ER test.

**Figure 4:**
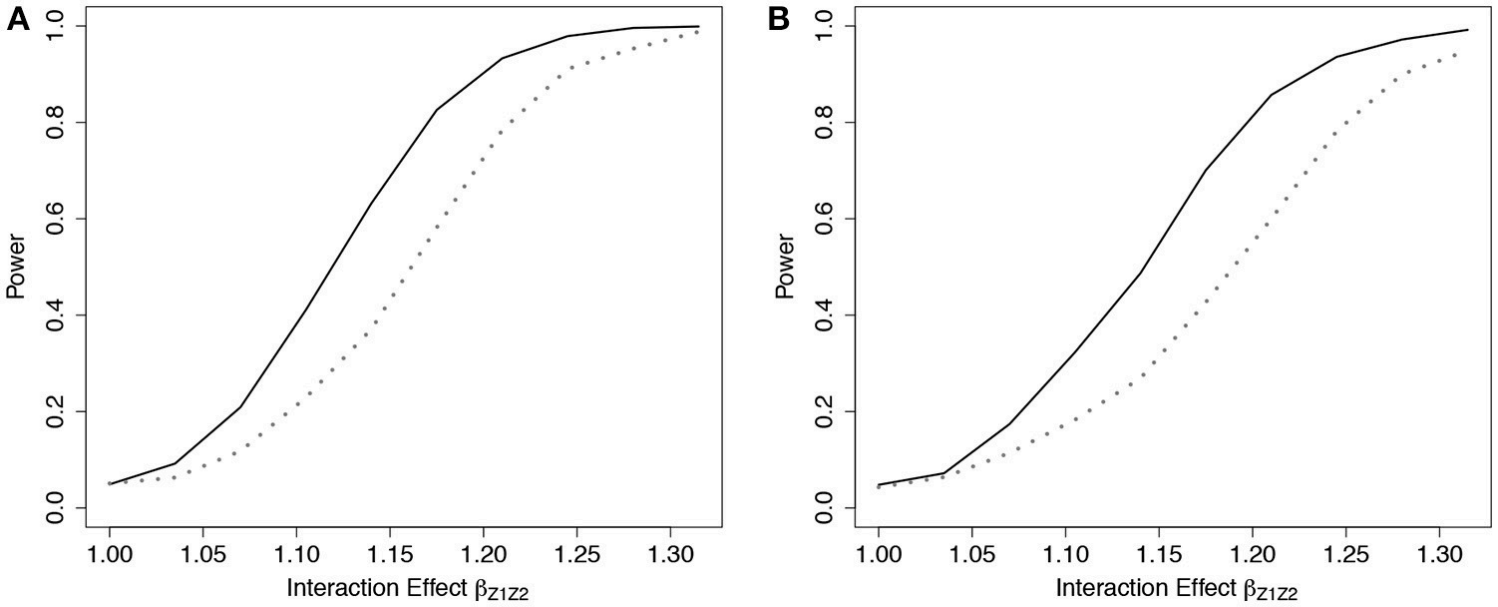
Power of tests under missing interaction models with Gaussian covariates *g*_03_. Power of the ER (solid line) and GB (dotted line) tests when interaction effect of *Z*_1_*Z*_2_ is missing from the fitted Cox model (3), using *g*_03_. *n* = 5,000 for the 2-covariate model with one interaction in the underlying model *g*_*A*3_, with either 0% **(A)** or 50% **(B)** lost to follow-up censoring. $Z_{1},Z_{2}\sim N\left(0,1\right)$.

To demonstrate the usefulness of the ER test beyond models using only genotypes as predictors, we considered scenarios involving covariates from a standard Gaussian distribution. For this scenario, we simulated data under (Equation 3) using *g*_*A*3_(**β**, ***Z***_*i*_) = β_*Z*_1__*Z*_*i*1_ + β_*Z*_2__*Z*_*i*2_ + β_*Z*_1_*Z*_2__*Z*_*i*1_*Z*_*i*2_, where $Z_{i1},Z_{i2}\sim \;N\left(0,1\right)$, and β_*Z*_*j*__ was fixed at log(1.15) for both covariates, and β_*Z*_1_*Z*_2__ was varied. We then fit a Cox model (Equation 3) that omitted the interaction term, using *g*_03_(**β**, ***Z***_*i*_) = β_*Z*_1__*Z*_*i*1_ + β_*Z*_2__*Z*_*i*2_, to examine the empirical power to detect the model misspecification. Similar to the SNP risk model examples, we expected that data simulated under *g*_*A*3_ and fit using a Cox PH model with *g*_03_ would show increased deviation in observed vs. expected survival at the extremes of the subject risk distribution (Web Figure 3 in the Supplementary Materials). The results showed a noticeable power increase in ER over GB for this scenario (Figure 4).

As demonstrated in Web Figure 3 in the Supplementary Materials, predicted risk can be highly sensitive to quadratic effects (e.g., interaction terms) of normally distributed covariates. As an example, patients with large negative values for both *Z*_1_ and *Z*_2_ will be predicted to have low risk from a fitted model with positive estimated main effects. However, if a large positive interaction effect is omitted from the model, the actual observed risk will be much higher resulting from the product of the two negative values. This contributes to the more significant improvement of ER over GB with Gaussian covariates compared to categorical ones.

### 3.3. Power of the ER test under misspecified models-additive covariate effects on the HR

Similar to Song et al. (2015), we examined the power of the ER test to detect model misspecification due to additive effects on the hazard. For this, we simulated data from (Equation 3) with $g_{A4}\left(\beta ,G_{i}\right)=log\left(1+\overset{p}{\underset{j=1}{\sum }}\beta_{G_{j}}G_{ij}\right)$; it's easy to see that this corresponds to additive effects for a fixed baseline hazard, *h*_0_(*t*). We then fit the corresponding Cox model for (Equation 3) with *g*_01_, which assumes a multiplicative effect of ***G*** on the hazard. We expected that the deviation from the assumed model of multiplicative effects on the HR would show increased deviation in observed vs. expected survival in the extremes of the subject risk distribution (Web Figure 4 in the Supplementary Materials).

We simulated data with a fixed β_*G*_*j*__ across all SNPs so that the marginal HR for each SNP in the fitted Cox model would range between 1 and 1.2, and between 1 and 1.4, for models with *p* = 10 SNPs and 5 SNPs, respectively. We specified α at 1, corresponding to a constant baseline hazard. Under each scenario, we fixed the event rate prior to 10 years at 20 and 50% in the absence of censoring.

**Figure 5:**
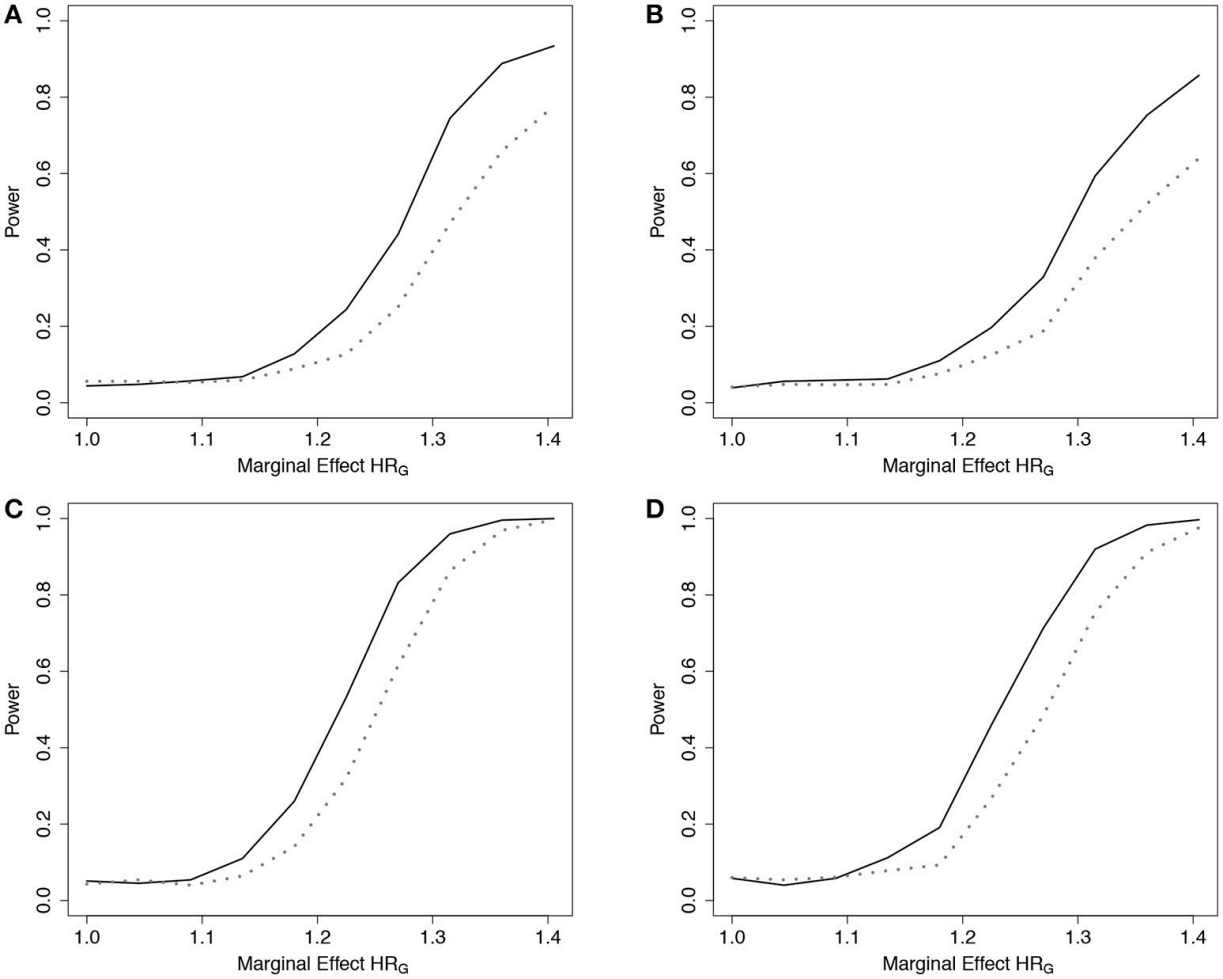
Power of tests under additive effect models with 5 SNPs. Power of the ER (solid line) and GB (dotted line) tests for detecting departures from the multiplicative model with 5 SNPs. Data simulated under additive effects on the HR, *g*_*A*4_, and fit using the multiplicative effects Cox model (3), with *g*_01_. *n* = 5,000 for the 5-SNP model with event rate 20% **(A**,**B)** or 50% **(C**,**D)**, and either 0% **(A**,**C)** or 50% **(B**,**D)** lost to follow-up censoring. *G*_*j*_ ~ *Bin*(2, 0.3).

Simulation results demonstrated that the ER was more powerful than the GB to detect departures from the multiplicative model when the true model was additive under the 5-SNP and 10-SNP models (Figure 5 and Web Figure 5 in the Supplementary Materials, respectively).

## 4. Application to cystic fibrosis-related diabetes

Cystic Fibrosis (CF) is a life-limiting recessive genetic disorder caused by mutations in the CF transmembrane conductance regulator (*CFTR*). CF affects multiple organs including the pancreas. As CF patients with severe *CFTR* mutations age, there is an increased risk of CF-related diabetes (CFRD), with a prevalence of 40% by the fourth decade of life (Moran et al., 2009). Uncontrolled CFRD is associated with muscle loss and declining lung function, which can be prevented by early detection and treatment. Models that predict CF individuals at high risk of developing CFRD could enable targeted surveillance programs with more frequent glucose monitoring so that CFRD is diagnosed early.

Multiple genetic factors, beyond *CFTR*, have been shown to contribute to CFRD including Type 2 diabetes susceptibility genes (Blackman et al., 2013) and CF-specific modifier genes (Li et al., 2014). Age-dependent predictive models (Heagerty et al., 2000) for CFRD based on genetic markers could be applied at birth to determine individuals at high risk, potentially benefiting the length and quality of life for individuals living with CF. However, if a CFRD risk model is well calibrated globally but poorly calibrated for the low or high risk groups, we question the utility of the model.

With CFRD event times based on data from the Canadian Cystic Fibrosis Gene Modifier Study we build and calibrate a predictive model for CFRD using a Cox PH model that includes as predictors six SNPs from six risk genes (coded additively) in addition to indicator variables for *CFTR* genotype severity and sex. Five of the genes (*SLC26A9, TCF7L2, CDKAL1, CDKN2A/B, IGF2BP2*) were previously identified in Blackman et al. (2013) and the sixth gene, *PRSS1*, encodes the enzyme cationic trypsinogen which is a biomarker of CFRD at birth (Soave et al., 2014).

**Figure 6:**
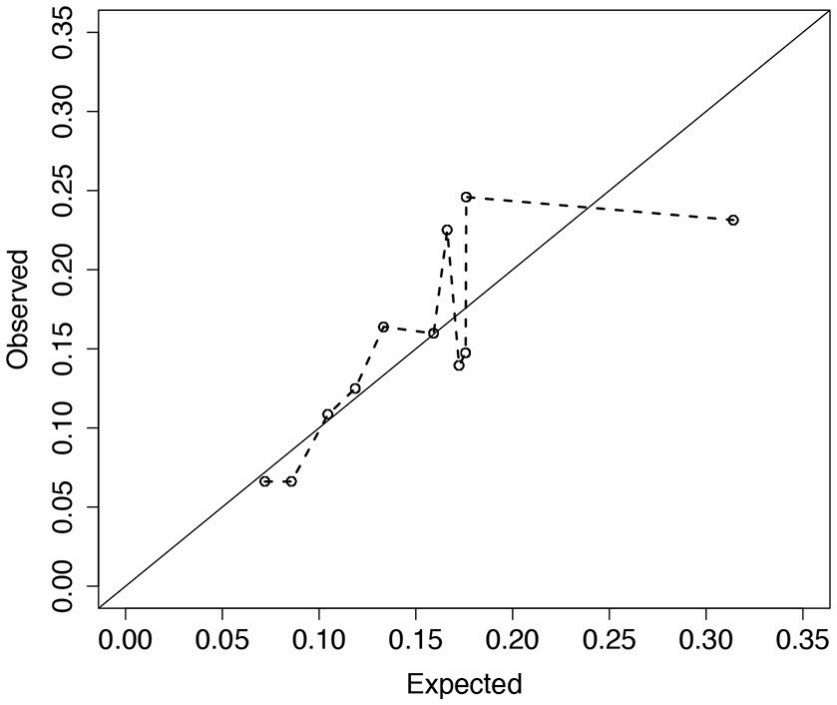
Observed vs. expected average number of CFRD events across 11 risk groups. Risk groups were defined according to the Cox PH model for CFRD onset risk with 8 covariates, ***z***_*i*_ (see section 4 for details). Expected number of events for each subject was calculated as the estimated cumulative hazard from the fitted Cox model with 8 covariates, ${\hat{\Lambda }}_{i}\left(x_{i}\right)=-log\left({\hat{S}}_{i}\left(x_{i}\right)\right)$, where ${\hat{S}}_{i}\left(x_{i}\right)={\hat{S}}_{0}{\left(x_{i}\right)}^{exp\left(z_{i}^{T}\hat{\beta }\right)}$. Observed number of events for each subject was calculated similarly using a Cox model with the risk-group indicator variables (***K***_*i*_) replacing the 8 covariates. Larger deviations from the diagonal line correspond to larger risk-group sums of martingales. Calibration test results for ER and GB were *p* = 0.047 and 0.21, respectively.

Analysis of 1,330 unrelated CF patients with complete information on the eight covariates is presented. Details of data collection, CFRD diagnosis, genotyping, and quality control procedures are reported elsewhere (Sun et al., 2012; Soave et al., 2014). Of the 1,330 included in the analysis, 203 patients had a CFRD diagnosis and the median age in years at last study visit (or diabetes) was 16.2 (21.6). To illustrate the fit of the model across the distribution of risks, we plot the observed vs. expected average absolute risk for each of 11 risk groups stratified according to their ordered expected risks (Figure 6). Ideally the points should cluster around the identity line, and if the model fits the data well no point should display a large deviation. However, for the groups with larger expected average risk, the deviations are large (Figure 6). For patients in the highest risk group, the model appears to over-estimate risk on average. The *p*-value for the ER test for this model is 0.047, whereas, the global GB test calculates a *p*-value = 0.2. This discrepancy is not surprising since ER is designed to have greater power than GB when the bias is greatest in the tails of the risk distribution.

The observed bias in risk-prediction for these patients, therefore, gives reason to reconsider the current model. A number of model fitting issues could contribute to the lack-of-fit in the tails, as we have demonstrated in this paper. Additional analyses involving interaction effects, scaling of covariates, and appropriateness of the multiplicative effects assumption should be considered.

## 5. Discussion

The Cox PH model for time-to-event data is straightforward to implement and does not require specification of a distribution for survival times. However, the Cox model does make several strong assumptions that may not be appropriate. Evaluation of a given model as a prediction tool requires assessment of both discrimination and calibration. Since the time and cost to obtain a second independent sample for model validation may be great, calibration assessment in a training sample can provide valuable information. Unfortunately, calibration is rarely reported (Collins et al., 2014).

The GOF test of Gronnesby and Borgan (1996) has been proposed as an omnibus test for global lack-of-fit assessment in Cox models. While this and other GOF methods have been shown to perform reasonably well as global tests, their power might be limited to detect bias of predicted risk at the extremes of the risk distribution where clinical decisions are generally made. Here, we proposed a new ER calibration test designed to examine the accuracy of risk predictions for patients at extreme (high or low) risk to be used alongside existing methods. Due to its construction, the distribution of the ER test statistic is intractable, and therefore we demonstrated a simulation method to estimate empirical significance that can be easily implemented using existing software.

Model misspecification can result in poorly calibrated risk estimates that are not detectable from standard GOF tests. Prediction tools that do not account for important interactions are likely to produce biased estimates of risk at the extreme tails of the population risk distribution, and these deviations should be detected more effectively through the proposed ER test. The simulation examples in section 3.2 indicate that the ER test could have increased power over the GB test to detect model misspecification when there are missing interactions. Although additive models may be a more natural starting point because they correspond to simple independent effects on the underlying risk factors (Weinberg, 1986), they are rarely implemented due to less convenient statistical properties. Similar to the logistic regression model (Song et al., 2015), model misspecification of the Cox model due to the assumption of multiplicative effects can also create bias at the extremes of the risk distribution. We observed that an incorrect multiplicative effects assumption in the Cox model can lead to dramatically underestimated survival probabilities for both extreme high and low risk groups when the underlying effects are additive (Web Figure 4 in the Supplementary Materials). In section 3.3, we showed that the ER test has advantages over the GB test in detecting the resulting bias.

We recognize that among competing calibration tests for time-to-event data, no single test will be most powerful for detecting lack-of-fit in all situations. Certainly, for model misspecification that results in bias over much of the range of estimated risks, the GB test should demonstrate greater power over a “max” test that simultaneously considers multiple subgroups of the data, such as the ER test. In section 3.2, we observed that the GB test slightly outperformed the ER test when very large interaction effect(s) were omitted from the working model but all main effects were included. This is likely because the larger interaction effects, when omitted, create deviations in predicted vs. observed risk over more of the risk distribution range, not just in the tails. In light of this, we recommend that our test should be thought of as a complementary tool when examining calibration of a risk model. Such an assessment should include visual inspection of a calibration plot similar to Figure 6 that can also be useful in explaining situations where the ER and GB tests give contradictory results.

Based on the convention for collapsing groups with fewer than 5 expected events, we found that collapsing was rarely required for the sample sizes of *n* = 5,000. For samples of *n* = 1,500 observations, collapsing occurred, however, it had little impact on type 1 error control (Table 2 and Web Table 3 in the Supplementary Materials). The power of the ER and GB tests compared with and without collapsing was also quite similar under most simulation models considered. Not surprisingly, for simulation models that resulted in frequent collapsing to fewer than 5 groups, there was a large decrease in power after applying the collapsing rule. Thus, for both type 1 error and power considerations, we recommend applying the ER test without collapsing, provided the sample size is no smaller than considered here, *n* = 1,500.

Our development of the ER test here focuses on detecting lack-of-fit for the purpose of internal model validation in a training dataset. Often, we need to evaluate the performance of a model in a new external cohort, where we might examine predicted survival probabilities. The implementation of both the GB and ER tests, however, only assesses accuracy of the linear predictor (estimated model coefficients) and does not incorporate information about the baseline hazard. As a result, these tests would be insensitive, in an external dataset, to detect any systematic bias (high or low) of predicted survival probabilities that require the baseline hazard estimated in the training dataset.

As the research community amasses new information about the molecular basis for disease, progress toward personalized medicine is being realized, revolutionizing disease treatment and preventative care. Accurate assessment of individual risk, incorporating both genetic and environmental factors plays a critical role in this initiative. Calibration tests such as the ER will become integral to risk-model determination to safeguard against biased risk-estimates for extreme risk patients, potentially most affected by clinical decisions.

## Ethics statement

This study was carried out in accordance with the recommendations of The Hospital for Sick Children, Research Ethics Board. The protocol was approved by the Research Ethics Board. All subjects gave written informed consent in accordance with the Declaration of Helsinki.

## Author contributions

DS and LS developed the method and wrote the manuscript. DS performed all analyses.

### Conflict of interest statement

The authors declare that the research was conducted in the absence of any commercial or financial relationships that could be construed as a potential conflict of interest. The reviewer JY and handling Editor declared their shared affiliation.
