# Supplementary material for: Testing Calibration of Cox Survival Models at Extremes of Event Risk
Source: Front Genet. 2018 May 22;9:177. doi: 10.3389/fgene.2018.00177 (PMC5972303; doi:10.3389/fgene.2018.00177)
Supplement: Supplementary file 1 [file Table_1.PDF]

Web-based Supplementary Materials for "Testing Calibration of Survival Models at Extremes of Event Risk"

by David Soave and Lisa J. Strug

CONTENTS

1 Web Appendix A: R code

2 Web Figures

3 Web Tables

1. Web Appendix A: R code

Although not directly available as output from the coxph() function in the "survival" R software package [R Core Team (2016). R: A language and environment for statistical computing. R Foundation for Statistical Computing, Vienna, Austria. URL https://www.R-project.org/], $\widehat{\Sigma}$ can be obtained as follows: (1) Fit a Cox model corresponding to model (Eq. 1) using coxph() to obtain estimates of the coefficients, $\widehat{\boldsymbol{\beta}}$. (2) Substitute these fixed estimates for $\boldsymbol{\beta}$ in a Cox model corresponding to model (Eq. 2) while also specifying $\boldsymbol{\gamma} = 0$; coefficients can be fixed in a coxph() fit by specifying *'iter.max=0'*. (3) Use the vcov() function to return the inverse of the observed information, $\widetilde{I}$, and obtain $\widehat{\Sigma}$ by taking the inverse of the submatrix with rows and columns corresponding to $\boldsymbol{\gamma}$. The following is example R code for this procedure.

```
############################################################
### Example R code for obtaining Σ̂ in the implementation of the ER and GB tests.
### See Section 2.3
cox0 <- coxph(Surv(time, event) ~ z)
cox1 <- coxph(Surv(time, event) ~ z+ K, iter.max=0,init=c(cox0$coef,rep(0,D-1)))
I.inv <- vcov(cox1)
Sigma.hat <- solve(I.inv[row.gamma,col.gamma])
############################################################
```

2. Web Figures

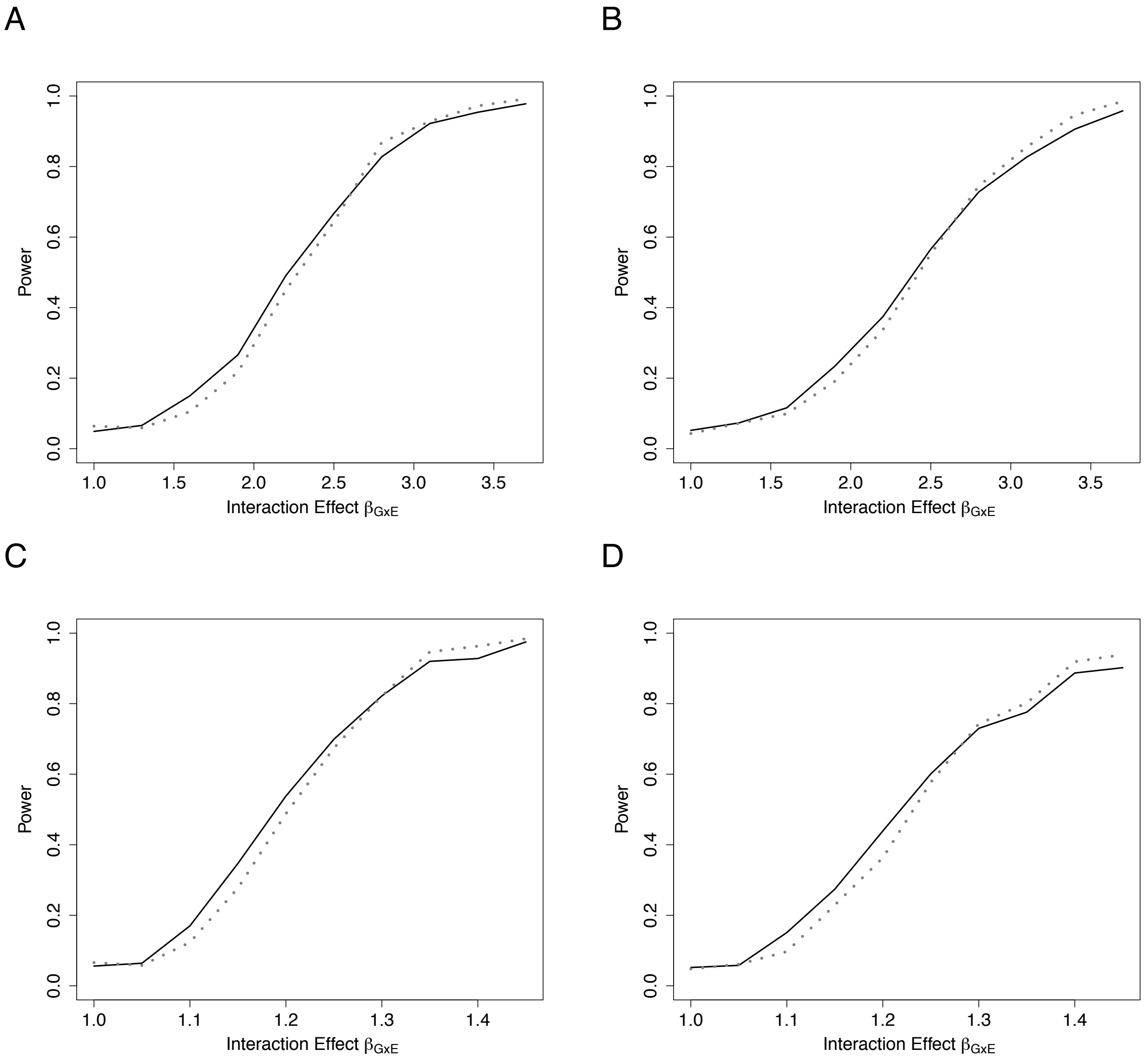

Figure 1. **Power of tests under missing interaction models** g_{02}**;** $n = 1500$**.** Power of the ER (solid line) and GB (dotted line) tests when $\boldsymbol{G}\text{x}E$ interaction terms are missing from the fitted Cox model (Eq. 3), but exposure E is included, using g_{02}. $n = 1500$ for the 10-SNP model with 1 interaction (**A** and **B**) or 10 interactions (**C** and **D**) in the underlying true model g_{A1}, and either 0% (**A** and **C**) or 50% (**B** and **D**) lost to follow-up censoring.

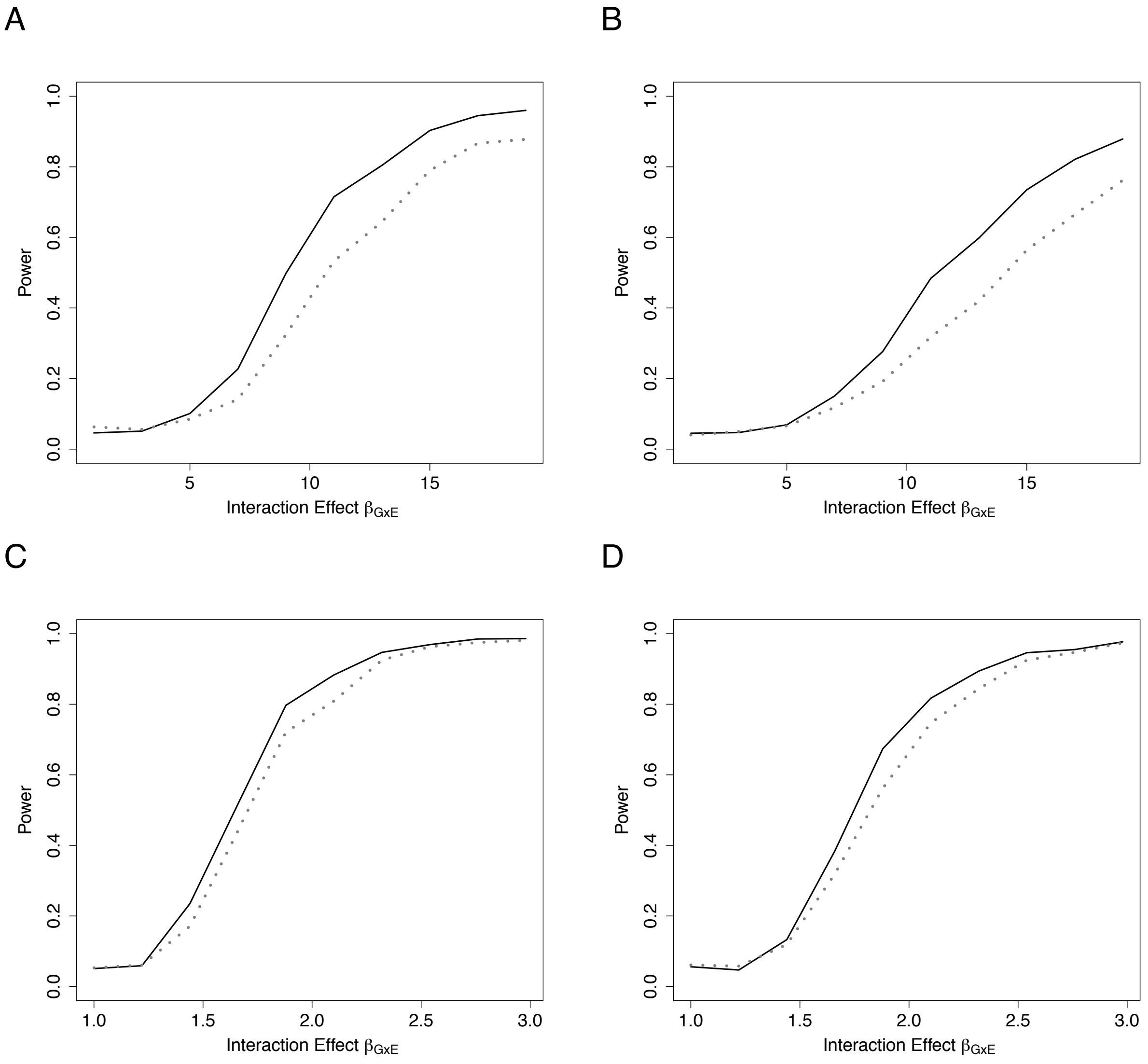

Figure 2. **Power of tests under missing interaction models g_{01}; $n = 1500$.** Power of the ER (solid line) and GB (dotted line) tests when main effect of E and $\boldsymbol{G}\text{x}E$ interaction terms are missing from the fitted Cox model (Eq. 3), using g_{01}. $n = 1500$ for the 10-SNP model with 1 interaction (**A** and **B**) or 10 interactions (**C** and **D**) in the underlying true model g_{A1}, and either 0% (**A** and **C**) or 50% (**B** and **D**) lost to follow-up censoring.

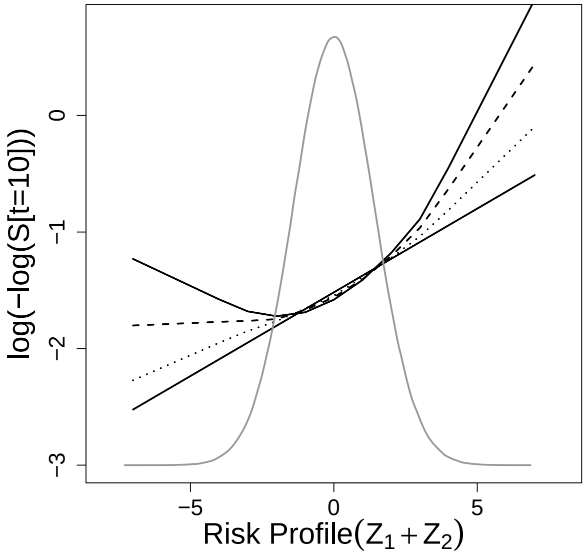

Figure 3. **log-log survival probability [$t = 10$ years] versus risk profile under missing interaction models with Gaussian covariates.** Time to event data were simulated under model (Eq. 3) with 2 standard Gaussian covariates (Z_1, Z_2) and an interaction ($g_{A3}(\boldsymbol{\beta}, \boldsymbol{Z}_i) = 1.15Z_1 + 1.15Z_2 + \beta_{Z_1Z_2}Z_1Z_2$ for $\beta_{Z_1Z_2} = 1.05$ (dotted line), 1.1 (dashed line) and 1.15 (curved solid line)). Cox models were fit using the null model $g_{02}(\boldsymbol{\beta}, \boldsymbol{Z}_i) = \beta_{Z_1}Z_{i1} + \beta_{Z_2}Z_{i2}$ to estimate survival probabilities at the administrative censoring time $t = 10$ years, ($\widehat{S}_i(t) = \widehat{S_0}(t)^{exp(\widehat{\beta}_{Z_1}Z_{i1}+\widehat{\beta}_{Z_2}Z_{i2})}$), which were then averaged by risk group (defined by nearest integer of risk profile, $Z_1 + Z_2$) (straight solid line - predicted survival). Observed survival probabilities (curved lines - observed survival) were obtained from a Cox model comparing patients in each risk group with the reference group. The reference group was the subset of individuals with risk profile $Z_1 + Z_2 = 0$. The distribution of subjects across the risk profile is shown as a density curve. Survival probabilities estimated for other time points ($t < 10$ years) yielded similar results.

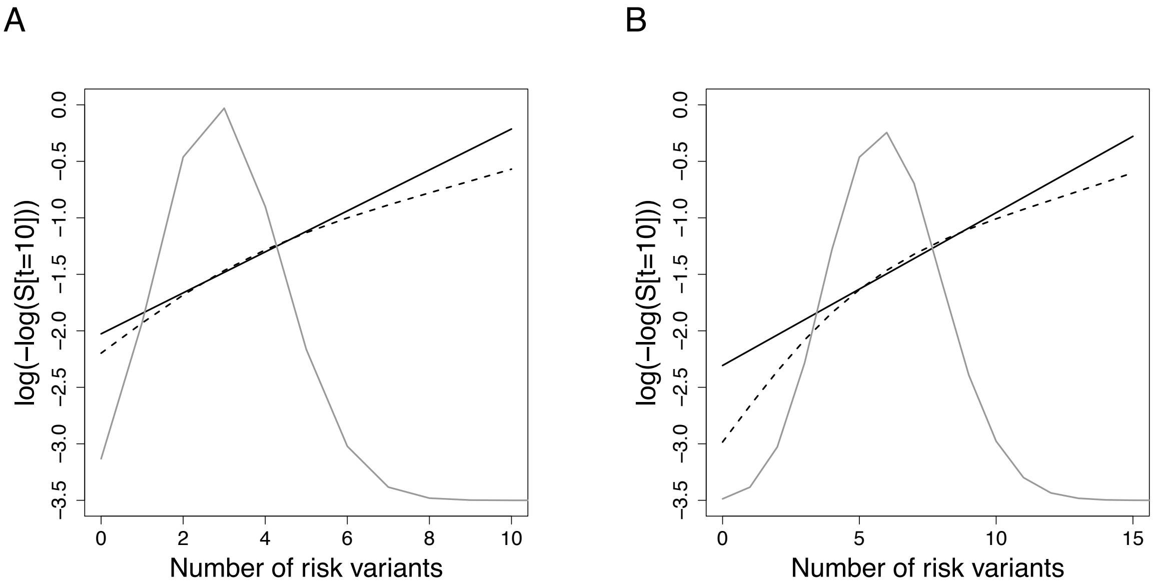

Figure 4. **log-log survival probability** [t = 10 **years**] **versus cumulative number of risk variants under additive effects models.** Time to event data were simulated under additive effects, model (Eq. 3), using $g_{A4}(\boldsymbol{\beta}, \boldsymbol{G}_i) = log(1 + \sum_{j=1}^{p} \beta_{G_j} G_{ij})$ with (**A**) $p = 5$ SNPs or (**B**) $p = 10$ SNPs (MAF=0.3 for each). Data were simulated with a fixed β_{G_j} across all SNPs so that the marginal HR for each SNP in the fitted Cox model was 1.15 and 1.2, for the 10-SNP and 5-SNP models, respectively. Cox models were fit, assuming multiplicative effects on the hazard, using the null model $g_{01}(\boldsymbol{\beta}, \boldsymbol{G}_i) = \sum_{j=1}^{10} \beta_{G_j} G_{ij}$ to estimate survival probabilities at time t=10 years ($\widehat{S}_i(t) = \widehat{S_0}(t)^{exp(\sum_{j=1}^{10} \widehat{\beta}_{G_j} G_{ij})}$), which were then averaged by risk group (defined by cumulative number of risk variants) (solid line - predicted survival). Observed survival probabilities (dashed line - observed survival) were obtained from a Cox model comparing patients in each risk group with the reference group. The reference group was the subset of individuals with 3 and 6 risk alleles for the 5-SNP and 10-SNP models respectively. The distribution of subjects per number of risk alleles is shown as a density curve. Survival probabilities estimated for other time points ($t < 10$ years) yielded similar results.

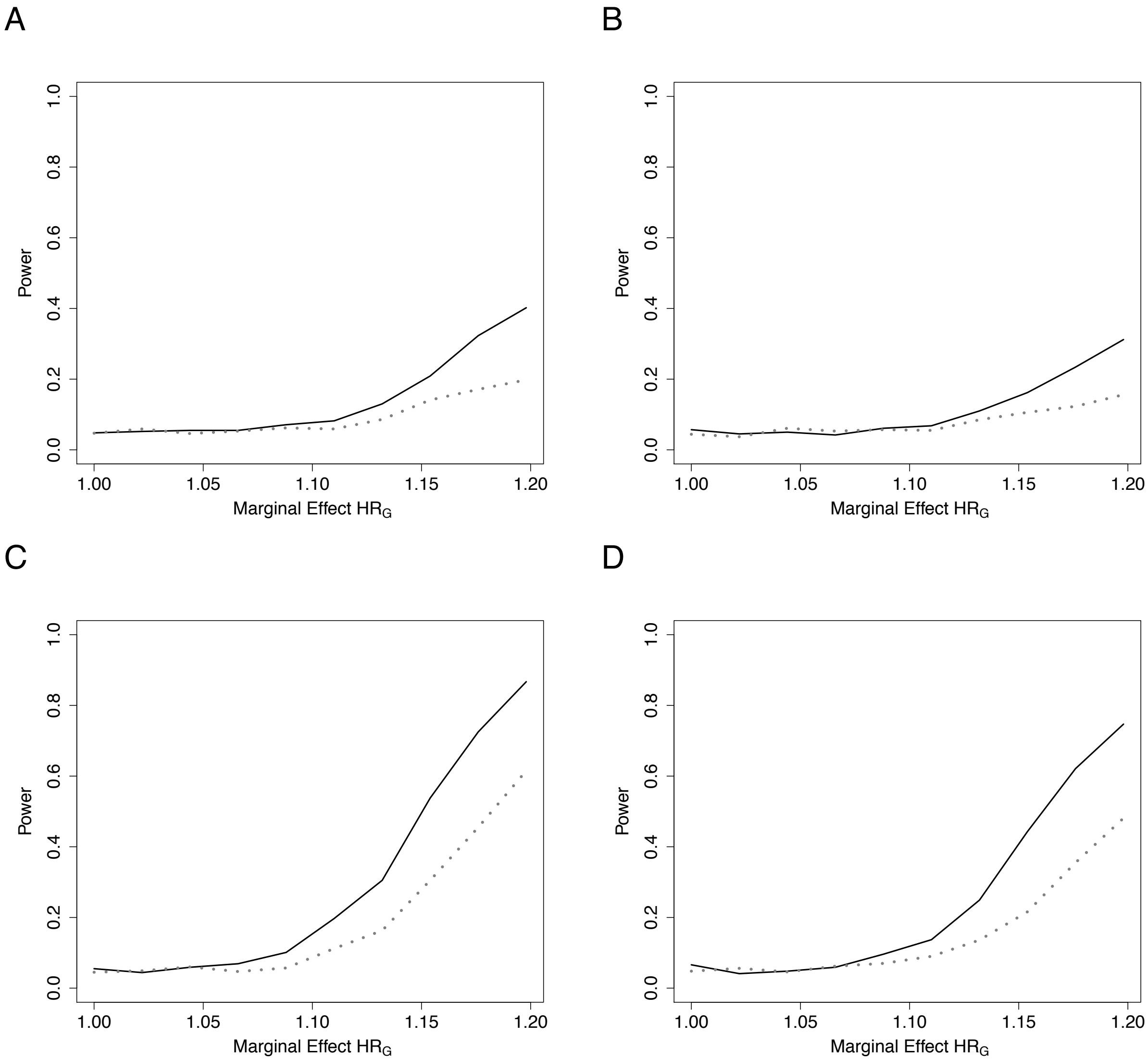

Figure 5. **Power of tests under additive effects models with 10 SNPs.** Power of the ER (solid line) and GB (dotted line) tests for detecting departures from the multiplicative model with 10 SNPs. Data simulated under additive effects on the HR, g_{A4}, and fit using the multiplicative effects Cox model (Eq. 3), with g_{01}. $n = 5000$ for the 10-SNP model event rate 20% (**A** and **B**) or 50% (**C** and **D**), and either 0% (**A** and **C**) or 50% (**B** and **D**) lost to follow-up censoring. $G_j \sim Bin(2, 0.3)$.

3. Web Tables

Table 1

Type-1 error of tests for decreasing baseline hazard. Event times simulated under the Weibull hazard model (Eq. 3) with g_{01} for a decreasing baseline hazard, $\alpha = 0.3$, with $p = 5$ or 10 covariates (genotypes). Empirical size is presented for the Gronnesby and Borgen test (GB) and the proposed Extreme Risk test (ER). The empirical type 1 error was estimated from 10,000 simulated replicates at the nominal 5% level.

	$p = 5$				$p = 10$			
	0% censoring		50% censoring		0% censoring		50% censoring	
Event Rate	GB	ER	GB	ER	GB	ER	GB	ER
$n = 5000$								
0.05	0.050	0.049	0.046	0.050	0.051	0.050	0.051	0.052
0.1	0.052	0.053	0.052	0.049	0.05	0.048	0.050	0.05
0.2	0.051	0.051	0.052	0.052	0.051	0.049	0.050	0.053
$n = 1500$								
0.05	0.053	0.053	0.049	0.053	0.052	0.048	0.050	0.048
0.1	0.053	0.049	0.052	0.052	0.057	0.052	0.053	0.053
0.2	0.049	0.052	0.049	0.050	0.058	0.051	0.053	0.052

Table 2

Type-1 error of tests for increasing baseline hazard. Event times simulated under the Weibull hazard model (Eq. 3) with g_{01} for a increasing baseline hazard, $\alpha = 3$, with $p = 5$ or 10 covariates (genotypes). Empirical size is presented for the Gronnesby and Borgen test (GB) and the proposed Extreme Risk test (ER). The empirical type 1 error was estimated from 10,000 simulated replicates at the nominal 5% level.

	$p = 5$				$p = 10$			
	0% censoring		50% censoring		0% censoring		50% censoring	
Event Rate	GB	ER	GB	ER	GB	ER	GB	ER
$n = 5000$								
0.05	0.052	0.049	0.053	0.052	0.049	0.046	0.051	0.048
0.1	0.050	0.053	0.055	0.054	0.051	0.050	0.053	0.053
0.2	0.051	0.050	0.049	0.050	0.051	0.049	0.054	0.053
$n = 1500$								
0.05	0.052	0.052	0.049	0.051	0.053	0.049	0.055	0.056
0.1	0.054	0.052	0.052	0.049	0.049	0.054	0.053	0.053
0.2	0.051	0.053	0.056	0.054	0.056	0.053	0.058	0.053

Table 3

Type-1 error of tests applying the 'no less than 5' convention for constant baseline hazard. Event times simulated under the Weibull hazard model (Eq. 3) with g_{01} for a constant baseline hazard, $\alpha = 1$, with $p = 5$ or 10 covariates (genotypes). Empirical size is presented for the Gronnesby and Borgen test (GB_{adj}) and the proposed Extreme Risk test (ER_{adj}). Both tests were applied to the augmented data following collapsing of groups based on the no less than 5 expected events per group convention. The empirical type 1 error was estimated from 10,000 simulated replicates at the nominal 5% level.

	$p = 5$				$p = 10$			
	0% censoring		50% censoring		0% censoring		50% censoring	
Event Rate	GB_{adj}	ER_{adj}	GB_{adj}	ER_{adj}	GB_{adj}	ER_{adj}	GB_{adj}	ER_{adj}
$n = 5000$								
0.05	0.049	0.049	0.050	0.049	0.053	0.056	0.054	0.045
0.1	0.050	0.052	0.053	0.049	0.054	0.051	0.052	0.054
0.2	0.052	0.049	0.052	0.05	0.048	0.051	0.052	0.050
$n = 1500$								
0.05	0.050	0.047	0.052	0.052	0.050	0.048	0.054	0.051
0.1	0.051	0.051	0.048	0.044	0.053	0.049	0.050	0.049
0.2	0.051	0.051	0.052	0.052	0.055	0.053	0.057	0.056
